# Sodium, potassium, and calories: implications for implementing a recommended diet in adults with hypertension

**DOI:** 10.1093/ajh/hpag021

**Published:** 2026-03-22

**Authors:** Michael G Buhnerkempe, Avani Yaganti, Stephanie Bitner, Zachary Settelmyer, Ananya Yaganti, Annah Carney, Vivek Prakash, Albert Botchway, Asad Cheema, John M Flack

**Affiliations:** Department of Internal Medicine, Southern Illinois University School of Medicine, Springfield, IL, United States; Department of Population Science and Policy, Southern Illinois University School of Medicine, Springfield, IL, United States; Center for Clinical Research, Southern Illinois University School of Medicine, Springfield, IL, United States; Department of Internal Medicine, Southern Illinois University School of Medicine, Springfield, IL, United States; Department of Internal Medicine, Southern Illinois University School of Medicine, Springfield, IL, United States; Department of Internal Medicine, Southern Illinois University School of Medicine, Springfield, IL, United States; Department of Internal Medicine, Southern Illinois University School of Medicine, Springfield, IL, United States; Department of Internal Medicine, Southern Illinois University School of Medicine, Springfield, IL, United States; Department of Internal Medicine, Southern Illinois University School of Medicine, Springfield, IL, United States; Center for Clinical Research, Southern Illinois University School of Medicine, Springfield, IL, United States; Department of Internal Medicine, Southern Illinois University School of Medicine, Springfield, IL, United States; Center for Clinical Research, Southern Illinois University School of Medicine, Springfield, IL, United States; Department of Internal Medicine, Southern Illinois University School of Medicine, Springfield, IL, United States; Department of Internal Medicine, Southern Illinois University School of Medicine, Springfield, IL, United States; Department of Population Science and Policy, Southern Illinois University School of Medicine, Springfield, IL, United States; Springfield Memorial Hospital, Springfield, IL, United States

**Keywords:** hypertension, dietary recommendations, life-style modification, sodium, potassium, blood pressure

## Abstract

**Background:**

Achieving evidence-based dietary recommendations for sodium and potassium are key lifestyle modifications for adults with hypertension. This study aimed to investigate how dietary recommendations have been followed in US adults with hypertension over time.

**Methods:**

Cross-sectional data on adults with hypertension was pooled from 12 National Health and Nutrition Examination Surveys (1999-2023). Dietary electrolyte intake was stratified as: (1) sodium ≤2300 mg/day (recommended) or ≤1500 mg/day (ideal), and (2) potassium ≥3500 mg/day (recommended) or ≥4700 mg/day (ideal).

**Results:**

From 1999 to 2023, 29.4% (95% confidence interval: 28.6-30.1) and 20.3% (19.6-21.0) of adults with hypertension met recommended sodium and potassium intakes, respectively, with 1.3% (1.1-1.5) meeting both levels. Adults with hypertension meeting ideal intakes for sodium, potassium, and both represented 9.5% (9.1-10.0), 6.1% (5.7-6.6), and 0.03% (0.01-0.06), respectively. The proportion meeting recommended thresholds for sodium has followed a U-shape over time reaching a minimum [24.3% (21.6-26.9)] in 2011-12 before increasing to 34.5% (31.3-37.7) in 2021-23 (*P* < .001). The proportion meeting the recommended potassium intake, however, has declined over time [yearly prevalence ratio = 0.99 (0.98-0.99), *P* < .001] reaching the lowest point [15.3% (13.5-17.0)] in 2021-23. The correlations between intakes of sodium and potassium, calories and sodium, and calories and potassium were 0.62, 0.77, and 0.71, respectively.

**Conclusions:**

A low percentage of adults with hypertension met both recommended sodium and potassium intakes with higher intakes of potassium associated with high sodium and calories.

## Graphical abstract

**Graphical Abstract:**
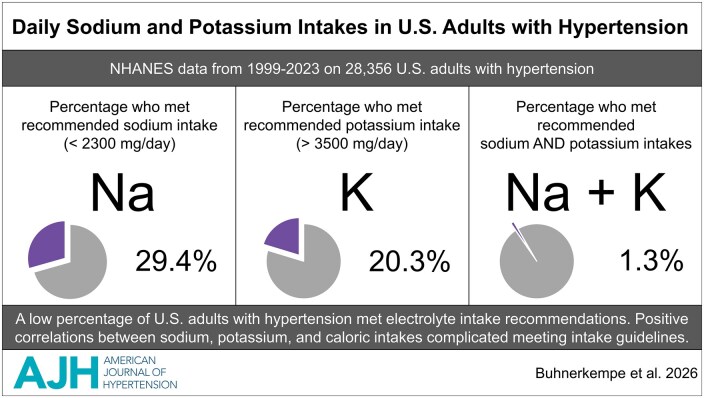

## Introduction

The current 2025 American Heart Association/American College of Cardiology (AHA/ACC) blood pressure guideline for Americans with hypertension recommends limiting sodium intake to below 2300 mg/day while maintaining potassium intake between 3500 and 5000 mg/day.^1^ A lower threshold for dietary sodium intake (<1500 mg/day) has been previously advanced.^1^^,^^2^ Similarly, a higher potassium threshold of >4700 mg/day has also been promoted.^3^^,^^4^ Previous population studies of data before 2014 have shown that individuals with hypertension rarely meet more stringent recommendations.^5^^,^^6^ However, it is unclear how the updated recommendations from 2025 have historically been followed in individuals with hypertension where dietary changes should be emphasized.

A decreased sodium-to-potassium ratio is known to reduce blood pressure.^7^ However, previous studies of individuals with hypertension have reported uptake of electrolyte recommendations independently,^5^^,^^6^ while acknowledging that the combined effect of sodium and potassium on blood pressure is greater than when either electrolyte is considered alone.^5^ Furthermore, sodium intake has been shown to increase with increased potassium intake complicating efforts to achieve low sodium/high potassium targets,^8^^,^^9^ as demonstrated by the 0.3% of the general US population meeting World Health Organization sodium-potassium goals.^9^ The intakes of these 2 dietary electrolytes must also be considered in the context of overall caloric intake as the relationship between sodium intake and blood pressure has been shown to be modified by energy intake.^10^ Accordingly, contemporary and historical diets of US adults with hypertension merit renewed scrutiny to gain insight into potential barriers to concurrently achieving updated recommended dietary intakes of sodium and potassium for those with hypertension.

In this study, we aim to determine how contemporary and historical diets in those with hypertension conform to current dietary recommendations for sodium and potassium intakes and how this has changed over time. We will also determine the correlation between the dietary intakes of sodium, potassium, and calories. Finally, we aim to identify demographic and dietary correlates associated with jointly meeting sodium and potassium recommendations to better understand potential strategies for meeting dietary guidelines. To accomplish this, we analyzed data from 12 cycles (1999-2023) of the National Health and Nutrition Examination Study (NHANES) covering US adults with hypertension.

## Methods

### Study data

NHANES is designed to capture a nationally representative sample of the noninstitutionalized US population to assess trends in health and nutrition. Informed consent was obtained from all participants, and protocols were approved by the National Center for Health Statistics’ (NCHS) Institutional Review Board.

We used data from 12 cycles conducted between 1999 and 2023, encompassing 119,555 participants. After exclusion, the subsequent analysis included 28,356 non-pregnant, adults with hypertension (Figure S1).

Data elements were standardized to ensure consistency across cycles. Individuals were eligible for two 24-hour dietary recall interviews, but not all participants completed both interviews. We chose to base total nutrient intakes on the first day only. An age of 80 years was assigned to those individuals over 80 years old. “Hispanic” was used to describe individuals with a race/ethnicity designation of “Mexican” or “Other Hispanic.” Blood pressure was averaged across all measures taken during an examination. Estimated glomerular filtration rate (eGFR) was calculated using the 2021 Chronic Kidney Disease Epidemiology Collaboration (CKD-EPI) equation.^11^^,^^12^ All other lab values were corrected according to NHANES specifications.

### Definitions

Sodium and potassium dietary intakes were categorized at 2 separate levels: (1) either recommended or not, and (2) either ideal or not. Recommended intake was defined according to the current 2025 AHA/ACC blood pressure guideline—sodium intake below 2300 mg/day and potassium intake above 3500 mg/day.^1^ Ideal intake for sodium was defined according to the 2025 AHA/ACC blood pressure guideline (<1500 mg/day).^1^^,^^2^ Ideal potassium intake was defined according to the 2015-2020 US Dietary Guideline (>4700 mg/day).^3^^,^^4^ Hypertension was defined as meeting 1 of 2 criteria: (1) blood pressure (BP) ≥130/80 mm Hg goal or (2) answering “yes” to “Are you now taking prescribed medication” for your high blood pressure/hypertension. Additional definitions for chronic kidney disease (CKD), albuminuria, prediabetes, and diabetes are provided in the Supplemental Methods.

### Statistical analysis

All analyses were weighted using the day 1 dietary recall weights to account for increased representation of weekend intakes and to provide unbiased estimates. All estimates were additionally age- and sex-standardized to the 2020 Census data.^13^ The proportion jointly meeting both sodium and potassium thresholds was compared to the expected proportion if sodium and potassium intake were independent (ie, the product of the proportion meeting the sodium threshold and the proportion meeting the potassium threshold) using a survey-weighted chi-squared test. Temporal analyses were done using survey-weighted Poisson or linear regressions with cycle as a covariate. The square of cycle was also considered in regression models to account for potential non-linearities through time. All other estimates were made across the entire 24-year period to improve reliability. Survey cycles were combined with updated weights constructed for the entire 24-year period according to the NCHS guideline.^13^ Comparisons between those who did and did not meet dietary sodium and potassium thresholds were done using survey-weighted chi-squared analyses for categorical variables and survey-weighted Wilcoxon rank sum tests for continuous variables. We also stratified the proportion of individuals meeting dietary intake goals on: (1) awareness of a hypertension diagnosis (ie, answering “Yes” to “Have you ever been told by a doctor or other health professional that you had hypertension, also called high blood pressure?”); (2) having controlled BP (<130/80 mm Hg); (3) taking antihypertension medications; and (4) hypertension defined using a BP threshold of 140/90 mm Hg. Missing values were not further evaluated or adjusted for because item non-response was low (<10%) for the variables considered, consistent with NCHS guideline.^13^ Analyses were conducted using the “survey” package^14^ in R statistical software.^15^

## Results

**Figure 1:**
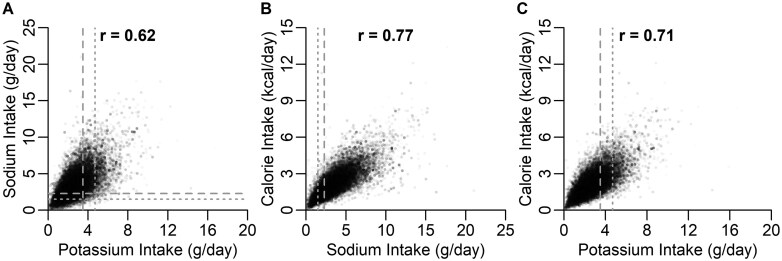
Relationship between daily (A) potassium and sodium intakes, (B) sodium and calorie intakes, and (C) potassium and calorie intakes. Dashed and dotted lines represent recommended and ideal intakes, respectively. Pearson correlation coefficients are provided.

Median [interquartile range (IQR)] sodium and potassium intakes were 3026 mg (2148-4213) and 2441 mg (1744-3279) per day, respectively. In 2021-23, median intakes were 2809 mg (2005-3966) and 2220 mg (1576-3009) of sodium and potassium, respectively. Higher sodium intake was associated with higher potassium intake (Figure 1A). Additionally, higher sodium and potassium intake were both associated with higher caloric intake (Figure 1B and C). The correlations between sodium and potassium (*r* = 0.02), sodium and calories (*r* = 0.21), and potassium and calories (*r* = 0.25) were all lower when only considering individuals meeting recommended intakes for both sodium and potassium.

Only 29.4% [95% confidence interval (28.6-30.1)] and 20.3% (19.6-21.0) of US adults with hypertension met recommended sodium and potassium intakes, respectively. Jointly meeting the recommended sodium and potassium thresholds was accomplished by only 1.3% (1.1-1.5), significantly below an expected 6.0% (determined by the product of proportions meeting sodium and potassium thresholds individually; *P < .*001). When considering the ideal thresholds, 9.4% (8.8-9.9) and 6.2% (5.7-6.7) met the lower sodium and higher potassium goals, respectively. Meeting both ideal intakes was accomplished by 0.03% (0.01-0.06), again significantly below an expected prevalence of 0.58% (*P < .*001).

**Figure 2:**
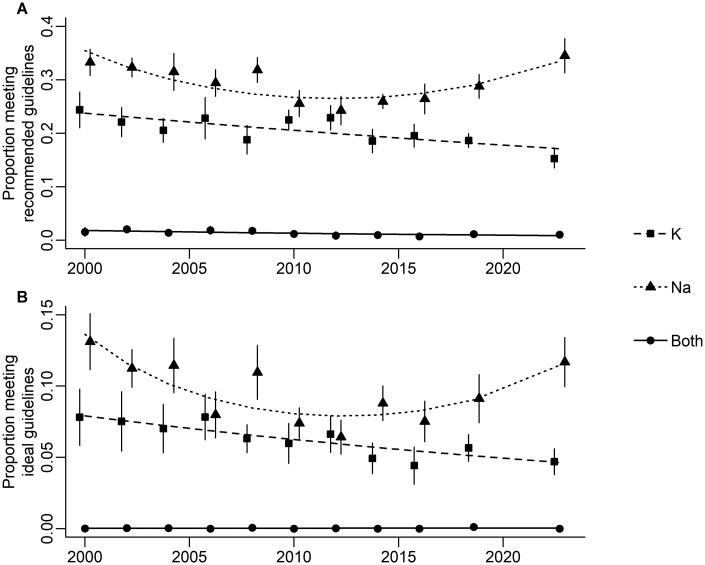
Proportion of US adults with hypertension who (A) met recommended thresholds for sodium (triangles), potassium (squares), or both (circles) and (B) met ideal thresholds for sodium, potassium, or both through time. Dashed, dotted, and solid lines depict the best fit linear or quadratic trend across years for potassium, sodium, and both, respectively. Points are placed at the interval mid-points for each cycle (eg, 2000.0 for the January 1999-December 2000 cycle, 2018.6 for the 3.2 year January 2017-March 2020 pre-pandemic cycle, and 2022.7 for the August 2021-August 2023 cycle).

Over time, the percentage of US adults with hypertension meeting recommended thresholds for sodium, potassium, and the combination of both ranged from 24.3% to 34.5%, 15.3% to 24.4%, and 0.7% to 2.0%, respectively (Figure 2A). The percentage meeting the recommended sodium intake showed a U-shape (*P < .*001; Table S1) reaching a minimum of 24.3% (95% confidence interval: 21.6-26.9) in 2011-12 before increasing to 34.5% (31.3-37.7) in 2021-23. The percentage meeting the recommended potassium intake decreased linearly through time reaching the lowest level [15.3% (13.5-17.0)] in 2021-23 [year-over-year prevalence ratio = 0.99 (0.98-0.99), *P < .*001; Table S2]. The percentage meeting both recommended intakes also declined through time [year-over-year prevalence ratio = 0.97 (0.95-0.99), *P = .*002; Table S3] reaching 1.0% (0.5-1.5) in 2021-23. The percentages meeting the ideal thresholds for sodium, potassium, and the combination of both ranged from 6.4% to 13.1%, 4.4% to 7.8%, and 0% to 0.12%, respectively, with 11.7% (9.9-13.4), 4.7% (3.8-5.6), and 0% meeting ideal intakes in 2021-23 (Figure 2B). Trends in ideal intakes through time largely mirrored those observed for recommended intakes (Tables S4-S6).

The mean ratio of sodium-to-potassium in the diet increased linearly over time from 1.32 [95% confidence interval (1.28-1.36)] in 1999-2000 to 1.43 (1.41-1.45) in 2021-23 (*P < .*001; Figure S2A; Table S7). Sodium density peaked in 2009-10 at 1730 mg/1000 calories (1702-1758) and has declined to 1607 mg/1000 calories (1577-1637) in 2021-23 (*P < .*001; Table S8) with all years having a mean sodium density above 1500 mg/1000 calories (Figure S2B). Similarly, potassium density peaked in 2009-10 at 1404 mg/1000 calories (1377-1430) and has declined to 1258 mg/1000 calories (1236-1281) in 2021-23 (*P < .*001; Figure S2C; Table S9).

Characteristics of individuals meeting recommended sodium and potassium levels, are shown in Table 1. Individuals who met both the recommended intakes were more likely to be White, had lower body mass index (BMI) and poverty levels, and lower prevalence of macroalbuminuria, prediabetes, diabetes, prior coronary heart disease (CHD), and stroke. Those meeting only the recommended sodium intake had the lowest caloric intake and the lowest levels of all dietary components (potassium, sodium, fat, protein, carbohydrates, cholesterol, and fiber). In contrast, those with only the recommended potassium intake had the highest caloric intakes and the highest levels of all dietary components. Meeting both recommended intakes resulted in more moderate caloric intakes and intakes of other dietary components.

**Table 1:** Characteristics of US adults with hypertension meeting recommended sodium (<2300 mg/day) and potassium (>3500 mg/day) intakes.

	Meets neither (51.7%)	Sodium only (28.1%)	Potassium only (19.0%)	Meets both (1.3%)	*P*
**Participant characteristics^a^**					
** Age, years**	56 (43-67)	62 (49-72)	53 (41-65)	61 (52-71)	<.001
** Sex—male**	53.1	34.4	76.4	46.9	<.001
** Race/Ethnicity**					<.001
** Black**	12.2	15.8	12.3	12.9	
** White**	66.4	60.0	71.6	74.5	
** Hispanic**	13.9	17.0	9.2	8.9	
** Other**	7.5	7.2	6.9	3.7	
** Systolic BP, mm Hg**	132.0 (121.7-142.3)	133.3 (122.7-146.0)	131.3 (122.0-140.7)	132.0 (122.7-144.0)	<.001
** Diastolic BP, mm Hg**	78.3 (68.7-84.7)	76.0 (66.0-83.7)	80.0 (70.7-85.3)	76.7 (67.7-84.0)	<.001
** BMI, kg/m^2^**	29.7 (25.8-34.5)	28.9 (25.2-33.6)	28.9 (25.6-33.5)	27.6 (24.1-31.1)	<.001
** Household income < $20,000**	20.8	28.8	17.4	18.6	<.001
** Education < high school**	17.3	25.6	15.0	16.3	<.001
** eGFR, mL/min/1.73 m^2^**	91.4 (75.6-102.9)	86.4 (68.6-98.7)	93.1 (79.5-104.5)	86.5 (73.1-98.8)	<.001
** Chronic kidney disease**					<.001
** Mild**	6.5	9.8	4.1	5.7	
** Severe**	3.2	5.9	1.4	2.9	
** Albuminuria**					<.001
** Micro**	12.1	14.5	10.3	12.7	
** Macro**	2.7	3.6	2.0	1.9	
** Prediabetes**	29.8	30.4	25.0	21.6	<.001
** Diabetes**	19.2	21.4	15.9	13.1	<.001
** Prior stroke**	4.7	7.2	4.0	3.8	<.001
** Prior CHD**	5.5	7.5	6.2	4.8	<.001
**Diet^a^**					
** Calories, kcal**	2008 (1638-2459)	1239 (950-1539)	2969 (2416-3716)	1903 (1585-2298)	<.001
** Potassium, mg**	2418 (1915-2896)	1641 (1183-2185)	4199 (3798-4908)	3907 (3674-4430)	<.001
** Sodium, mg**	3353 (2787-4209)	1725 (1359-2035)	4784 (3730-6292)	1997 (1713-2147)	<.001
** Fat, g**	80.0 (60.0-104.0)	43.0 (30.0-57.7)	114.8 (83.7-153.8)	59.7 (40.6-85.7)	<.001
** Protein, g**	75.8 (60.4-95.1)	44.6 (32.8-57.3)	117.4 (93.6-148.3)	79.1 (61.2-92.9)	<.001
** Carbohydrates, g**	229.2 (176.8-295.5)	155.0 (111.7-205.4)	341.1 (271.0-437.8)	260.0 (212.6-316.9)	<.001
** Cholesterol, mg**	248.0 (157.0-406.0)	121.4 (72.0-208.0)	364.0 (224.5-573.0)	159.0 (101.0-255.0)	<.001
** Fiber, g**	13.9 (9.9-18.7)	9.3 (6.0-13.8)	24.5 (18.4-32.6)	23.1 (17.4-29.6)	<.001

a Estimates are either % for categorical variables or median (25th percentile, 75th percentile) for continuous variables.

**Figure 3:**
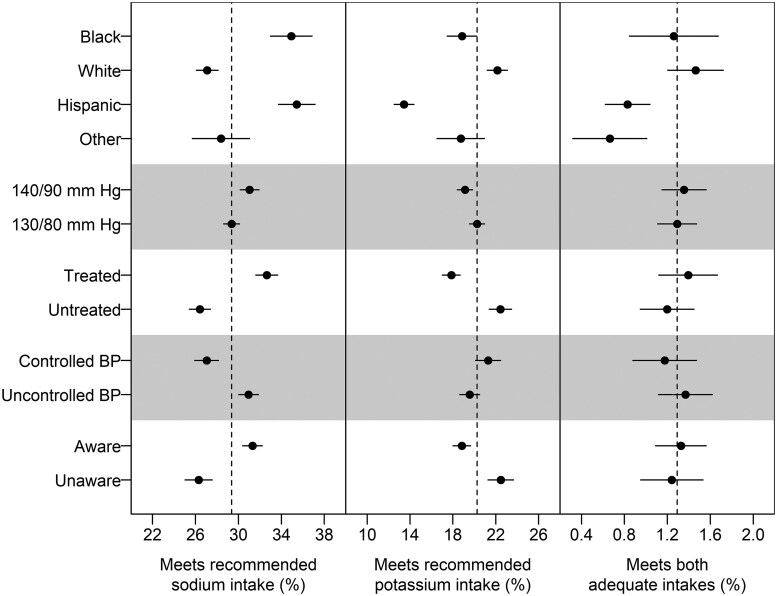
Forest plot showing the impact of awareness of a hypertension diagnosis (aware vs. unaware), BP control (controlled BP vs. uncontrolled BP), hypertension treatment (treated vs. untreated), hypertension definition (140/90 mm Hg vs. 130/80 mm Hg), and race (black vs. White vs. Hispanic vs. Other) on the percentage of individuals with hypertension meeting (from left to right) recommended sodium, potassium, and both thresholds. Horizontal bars depict 95% confidence intervals with dashed lines showing the overall prevalence using a hypertension definition of 130/80 mm Hg.

Effects of race and factors related to hypertension diagnosis, treatment, and control on meeting recommended sodium and potassium intakes are shown in Figure 3. Individuals who were aware of their hypertension were more likely to meet recommended sodium intake goals but were less likely to meet the recommended potassium intake compared to those who were unaware of their hypertension. Compared to those with controlled BP, those with uncontrolled BP were more likely to meet the recommended sodium intake and less likely to meet the recommended potassium intake. Untreated hypertension, compared to treated hypertension, was associated with decreased and increased likelihood of meeting recommended sodium and potassium intakes, respectively. Using a 140/90 mm Hg threshold vs. 130/80 mm Hg to define hypertension resulted in more individuals with hypertension who met recommended sodium intakes and fewer meeting recommended potassium intakes. Racial differences in meeting recommended sodium, potassium, and both sodium and potassium intakes were pronounced but not necessarily consistent. The correlations between sodium, potassium, and calories were largely consistent across race/ethnicity (Figures S3-S6).

## Discussion

Our study demonstrates that adults with hypertension in the US do not often consume recommended amounts of sodium or potassium put forward by the 2025 AHA/ACC blood pressure guideline^1^ and rarely meet both recommendations. The proportion of adults who consumed the recommended amount of sodium initially declined before increasing in recent years, while the proportion meeting recommended intakes of potassium and both electrolytes declined over the period covered in this study, 1999-2023. Additionally, potassium, sodium, and caloric intake are all strongly correlated making a high potassium, low salt, low calorie diet difficult to achieve. Individuals meeting recommended levels of intake of both electrolytes generally had healthier diets than those meeting recommended intakes of only one electrolyte. These individuals managed to break the associations between sodium, potassium, and calories with lower correlations between these dietary components. While most Americans do not meet the recommendations reviewed in this study, White race, lower BMI, and lower poverty level were associated with achieving both recommended sodium and potassium intake.

The strongest clinical factor associated with meeting dietary guidelines was treatment with anti-hypertensive medications. Treated individuals had better adherence to sodium recommendations. Strong correlations between higher sodium and higher potassium and higher calorie intakes likely explain why these same individuals are less adherent to the potassium guideline. Our data suggest that reductions in sodium for most individuals was primarily achieved through a reduction in caloric intake rather than a true shift in diet. Treatment likely coincides with awareness of a hypertension diagnosis, which showed similar patterns in both sodium and potassium intakes. Given that a BP goal of 130/80 mm Hg was only introduced in 2017 in the later stages of these NHANES cycles, it is also likely that differences in recommended sodium and potassium intakes between hypertension definitions can be attributed to awareness differences. Although considerable racial differences in achieving recommended sodium and potassium intakes were observed, correlations between sodium, potassium, and calories were consistent indicating that modifying contemporary diets to meet both electrolyte intakes simultaneously will be difficult, regardless of race.

Our results also align with previously published estimates of the percentage of adults with hypertension that met recommended sodium intakes (27.32%).^5^ On the contrary, our study found that 6.2% of US adults with hypertension met the ideal potassium guideline (>4700 mg/day) compared to 1% and 2.61% in other studies.^5^^,^^6^ These studies used 2 days of dietary recall, while we opted for one to increase sample sizes. Although dietary intakes are similar between days, caloric intake is marginally higher on day 1 which might account for some of this observed difference.^16^ Also, these studies excluded patients on anti-hypertensive medications, which may have played a role in the difference.^5^^,^^6^

Differences in education and income levels between those who meet recommended intakes highlight the role social determinants of health likely play in meeting recommended intakes. Social factors dictate access to fresher, healthier foods,^17^ and efforts to improve access will be key to improving dietary outcomes. More consistent embedding of advocates, such as community health workers, in hypertension care teams will be needed to address this barrier. Additionally, our results demonstrate that meeting both sodium and potassium intake recommendations is complex requiring choices that improve diet quality rather than simply reducing caloric intake. Specifically, an emphasis on sodium and potassium density in foods could help to highlight healthier choices. Although sodium density in the diets of US adults with hypertension has decreased in recent years, even on a 1000 calorie diet, the average American with hypertension will not meet the ideal sodium intake. This may reflect recent work that shows decreases in the sodium content of new food items from US chain restaurants, despite high sodium content overall and unchanged sodium content across all menu items.^18^^,^^19^ Potassium density has also decreased yielding diets that are relatively enriched in sodium relative to potassium. Although food preparation is known to reduce potassium content,^20^ whether these changes reflect changes in habits or the underlying food supply is unclear. Overall, navigation of these complexities within a changing food landscape will require improved education and support for individuals with hypertension to promote healthy, sustainable dietary changes. As advanced in the 2025 AHA/ACC guideline,^1^ dieticians and community health workers embedded in multidisciplinary hypertension care teams will likely be instrumental to achieving these goals.

Low adherence to potential dietary changes has been a limitation of such approaches in the past.^21^ The dietary approaches to stop hypertension (DASH) diet has been shown to reduce systolic and diastolic blood pressure.^22^ The 2025 AHA/ACC hypertension guideline shares the same sodium intake thresholds as the DASH diet. The DASH diet has a higher potassium intake goal (>4700 mg/day, equivalent to the ideal level used here) than the 2025 AHA/ACC guideline and emphasizes a holistic approach to lowering blood pressure through the consumption of lean proteins, fruits, whole grains, and foods low in unsaturated fats.^23^ A systematic review showed a correlation between increased adherence to the DASH diet and decreased cardiovascular disease mortality.^24^ However, these studies primarily used feeding trials^25^^,^^26^ where food is provided compared to clinical settings where participants only receive written and verbal guidance. Indeed, monetary support for self-directed shopping for DASH groceries has been shown to be less effective than curated home deliveries provided by a study team at lowering blood pressure and sodium intake,^27^ again emphasizing the complexities in adhering to diets like DASH that promote both recommended sodium and potassium intakes. Further research to understand how to increase adherence to established diets like DASH that promote this guideline is needed.

Limitations of this study include using dietary recall, which is known to underestimate sodium, potassium, and caloric intake.^28^ The data also does not include information on dietary supplements, a potential source of electrolyte and calorie intake. Furthermore, recommended intakes were defined using the 2025 AHA/ACC hypertension guideline; however, the study data in NHANES is from 1999 to 2023. This temporal disconnect means our results may be less applicable to current individuals with stage 1 hypertension who may only receive lifestyle counseling unless they are either deemed “high risk” or remain uncontrolled after 3-6 months of diet/lifestyle intervention. Sensitivity analyses using a hypertension definition of 140/90 mm Hg showed a significant, but not large, difference at this higher threshold indicating that this impact is likely low. However, future cycles of NHANES will provide insight on the number of participants meeting the updated guideline since their implementation. Finally, as the study data ends in 2023, it does not account for longer-term changes in dietary trends after the COVID-19 pandemic. Americans have experienced an overall decline in nutritional status since the COVID-19 pandemic,^29^ and the resultant effect on sodium and potassium intake in adults with hypertension is uncertain.

In summary, we found that a low percentage of US adults with hypertension have met current recommendations for sodium and potassium intake, and the percentage meeting potassium recommendations has decreased over time with some positive momentum in meeting sodium targets in recent years. These results show that individuals are not simply trying and failing to meet relatively unattainable thresholds, but that they fail to even meet the relatively more modest recommendations in the current guideline. Our results also point to 2 key barriers to achieving both healthy sodium and potassium intakes: (1) the complexities in breaking the strong ties between sodium, potassium, and calories that are likely driven by processed foods and (2) social determinants of health that likely limit access to fresh foods.

## Supplementary Material

hpag021_Supplementary_Data

## Data Availability

Data is publically available and can be downloaded from the National Health and Examination Survey (NHANES) website: https://www.cdc.gov/nchs/nhanes/.
