# Supplementary material for: Sodium, potassium, and calories: implications for implementing a recommended diet in adults with hypertension
Source: Am J Hypertens. 2026 Mar 22;39(8):997–1004. doi: 10.1093/ajh/hpag021 (PMC13369970; doi:10.1093/ajh/hpag021)
Supplement: hpag021_Supplementary_Data [file hpag021_supplementary_data.docx]

**Supplement Information For:**

Sodium, Potassium and Calories: Implications for Implementing a Recommended Diet in Adults with Hypertension.

Michael G. Buhnerkempe^1,2,3^, Avani Yaganti^1^, Stephanie Bitner^1^, Zachary Settelmyer^1^, Ananya Yaganti^1^, Annah Carney^1^, Vivek Prakash^1,3^, Albert Botchway^1,3^, Asad Cheema^1^, John M. Flack^1,2^

*^1^Department of Internal Medicine, Southern Illinois University School of Medicine, Springfield, IL*

*^2^Department of Population Science and Policy, Southern Illinois University School of Medicine, Springfield, IL*

*^3^Center for Clinical Research, Southern Illinois University School of Medicine, Springfield, IL*

**Supplemental Methods**

*Definitions*

Chronic kidney disease (CKD) was defined as mild or severe when eGFR was 45-60 or <45 mL/min/1.73m^2^, respectively. Albuminuria was defined using the urine albumin to creatinine ratio (mg/g) as follows: normal (< 30), microalbuminuria (≤ 30 and < 300), or macroalbuminuria (≥ 300). Individuals were said to have pre-diabetes if they answered “no” to “Have you ever been told by a doctor or health professional that you have diabetes?” and either (1) having hemoglobin A1c (HbA1c) between 5.7 and 6.4% without a prescription for a diabetes medication other than metformin or (2) having a prescription for metformin with HbA1c below 5.7^1,2^. Diabetes was defined by meeting any of 3 criteria: (1) answered “yes” to “Have you ever been told by a doctor or health professional that you have diabetes?”; (2) were prescribed one or more diabetes medications; or (3) were not prescribed diabetes medications but had a hemoglobin A1C 6.5% or higher^1^.

*References*

1. American Diabetes A. 2. Classification and Diagnosis of Diabetes: Standards of Medical Care in Diabetes-2018. *Diabetes Care* 2018; **41**(Suppl 1)**:** S13–S27.

2. American Diabetes A. 5. Prevention or Delay of Type 2 Diabetes: Standards of Medical Care in Diabetes-2018. *Diabetes Care* 2018; **41**(Suppl 1)**:** S51–S54.

**Supplemental Tables**

Table S1: Linear and polynomial model coefficients for survey-weighted Poisson regressions showing the relationship between number meeting recommended sodium intake and NHANES cycle year.

|  | **Linear Model** | | **Non-linear Model** | |
| --- | --- | --- | --- | --- |
| **Term** | **Coefficient** | **p-value** | **Coefficient** | **p-value** |
| Intercept | 4.65 | 0.28 | 8183 | <0.001 |
| Year | -0.003 | 0.18 | -8.14 | <0.001 |
| Year^2^ | - | - | 0.002 | <0.001 |

Table S2: Linear and polynomial model coefficients for survey-weighted Poisson regressions showing the relationship between number meeting recommended potassium intake and NHANES cycle year.

|  | **Linear Model** | | **Non-linear Model** | |
| --- | --- | --- | --- | --- |
| **Term** | **Coefficient** | **p-value** | **Coefficient** | **p-value** |
| Intercept | 27.52 | <0.001 | -2835 | 0.09 |
| Year | -0.014 | <0.001 | 2.83 | 0.09 |
| Year^2^ | - | - | -0.001 | 0.09 |

Table S3: Linear and polynomial model coefficients for survey-weighted Poisson regressions showing the relationship between number meeting both recommended sodium and potassium intakes and NHANES cycle year.

|  | **Linear Model** | | **Non-linear Model** | |
| --- | --- | --- | --- | --- |
| **Term** | **Coefficient** | **p-value** | **Coefficient** | **p-value** |
| Intercept | 61.86 | 0.004 | 4691 | 0.47 |
| Year | -0.032 | 0.002 | -4.64 | 0.48 |
| Year^2^ | - | - | 0.001 | 0.48 |

Table S4: Linear and polynomial model coefficients for survey-weighted Poisson regressions showing the relationship between number meeting ideal sodium intake and NHANES cycle year.

|  | **Linear Model** | | **Non-linear Model** | |
| --- | --- | --- | --- | --- |
| **Term** | **Coefficient** | **p-value** | **Coefficient** | **p-value** |
| Intercept | 15.70 | 0.07 | 14310 | <0.001 |
| Year | -0.009 | 0.04 | -14.22 | <0.001 |
| Year^2^ | - | - | 0.004 | <0.001 |

Table S5: Linear and polynomial model coefficients for survey-weighted Poisson regressions showing the relationship between number meeting ideal potassium intake and NHANES cycle year.

|  | **Linear Model** | | **Non-linear Model** | |
| --- | --- | --- | --- | --- |
| **Term** | **Coefficient** | **p-value** | **Coefficient** | **p-value** |
| Intercept | 44.50 | <0.001 | 987.5 | 0.74 |
| Year | -0.02 | <0.001 | -0.96 | 0.75 |
| Year^2^ | - | - | 0.0002 | 0.76 |

Table S6: Linear and polynomial model coefficients for survey-weighted Poisson regressions showing the relationship between number meeting both ideal sodium and potassium intakes and NHANES cycle year.

|  | **Linear Model** | | **Non-linear Model** | |
| --- | --- | --- | --- | --- |
| **Term** | **Coefficient** | **p-value** | **Coefficient** | **p-value** |
| Intercept | -91.77 | 0.35 | -4562 | 0.76 |
| Year | 0.04 | 0.39 | 4.49 | 0.76 |
| Year^2^ | - | - | -0.001 | 0.76 |

Table S7: Linear and polynomial model coefficients for survey-weighted linear regressions showing the relationship between sodium-to-potassium ratio and NHANES cycle year.

|  | **Linear Model** | | **Non-linear Model** | |
| --- | --- | --- | --- | --- |
| **Term** | **Coefficient** | **p-value** | **Coefficient** | **p-value** |
| Intercept | -9.49 | <0.001 | -300.4 | 0.54 |
| Year | 0.005 | <0.001 | 0.29 | 0.54 |
| Year^2^ | - | - | -7.19 x 10^-5^ | 0.55 |

Table S8: Linear and polynomial model coefficients for survey-weighted linear regressions showing the relationship between sodium density and NHANES cycle year.

|  | **Linear Model** | | **Non-linear Model** | |
| --- | --- | --- | --- | --- |
| **Term** | **Coefficient** | **p-value** | **Coefficient** | **p-value** |
| Intercept | -2249 | 0.46 | 3.00 x 10^6^ | <0.001 |
| Year | 1.95 | 0.20 | 2983 | <0.001 |
| Year^2^ | - | - | -0.74 | <0.001 |

Table S9: Linear and polynomial model coefficients for survey-weighted linear regressions showing the relationship between potassium density and NHANES cycle year.

|  | **Linear Model** | | **Non-linear Model** | |
| --- | --- | --- | --- | --- |
| **Term** | **Coefficient** | **p-value** | **Coefficient** | **p-value** |
| Intercept | 11450 | <0.001 | -1.46 x 10^6^ | <0.001 |
| Year | -5.05 | <0.001 | 1461 | <0.001 |
| Year^2^ | - | - | -0.36 | <0.001 |

**Supplemental Figures**

**
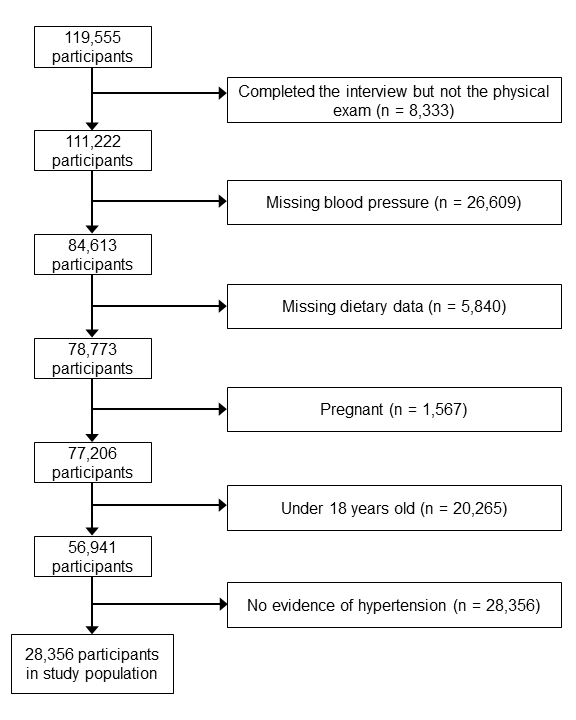
**

Figure S1: Flowchart for participant inclusion/exclusion.


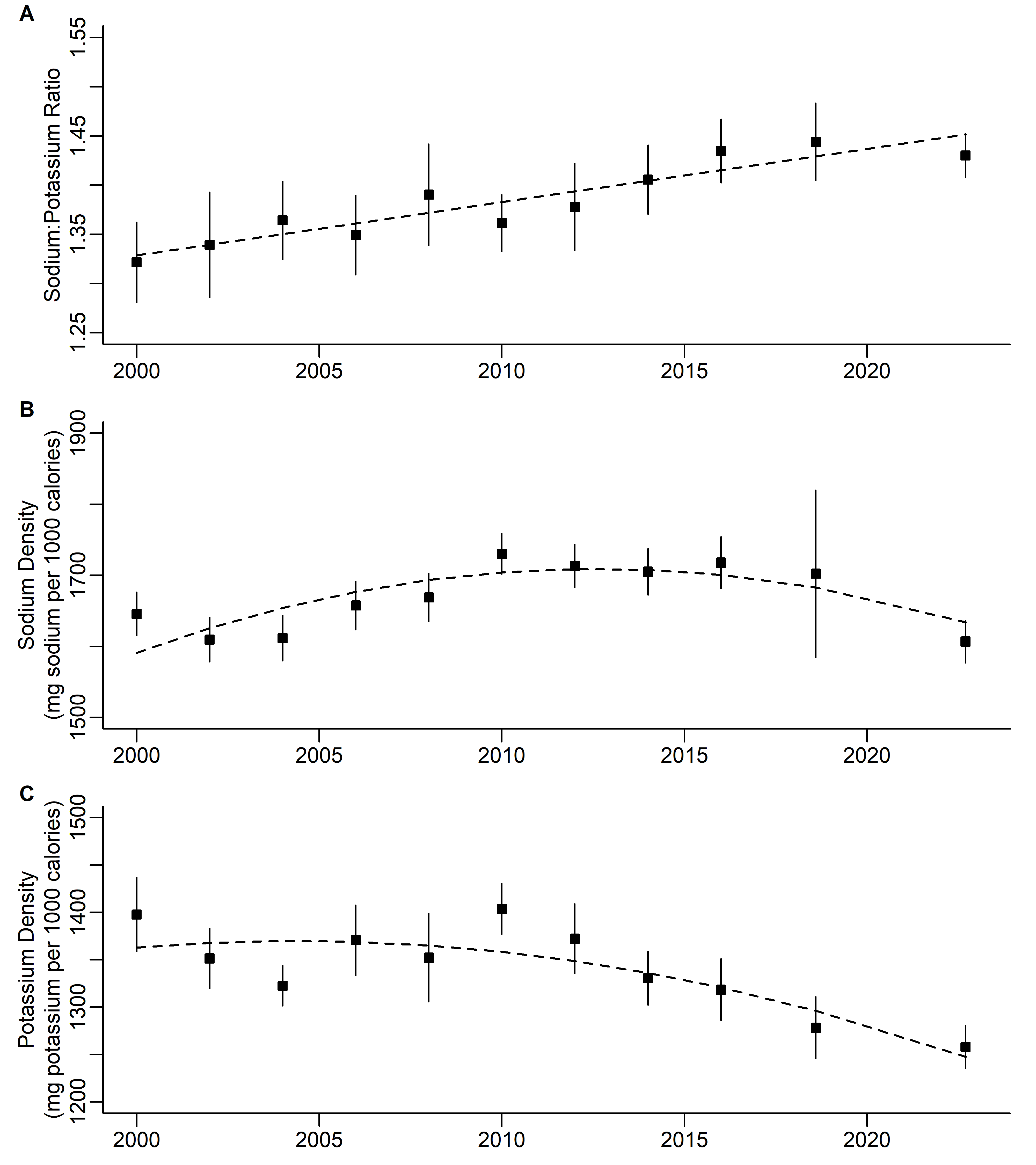


Figure S2: Mean (A) sodium-to-potassium ratio, (B) sodium density, and (C) potassium density in the diets of U.S. adults with hypertension through time. Dashed lines depict the best fit linear or polynomial trend across years.


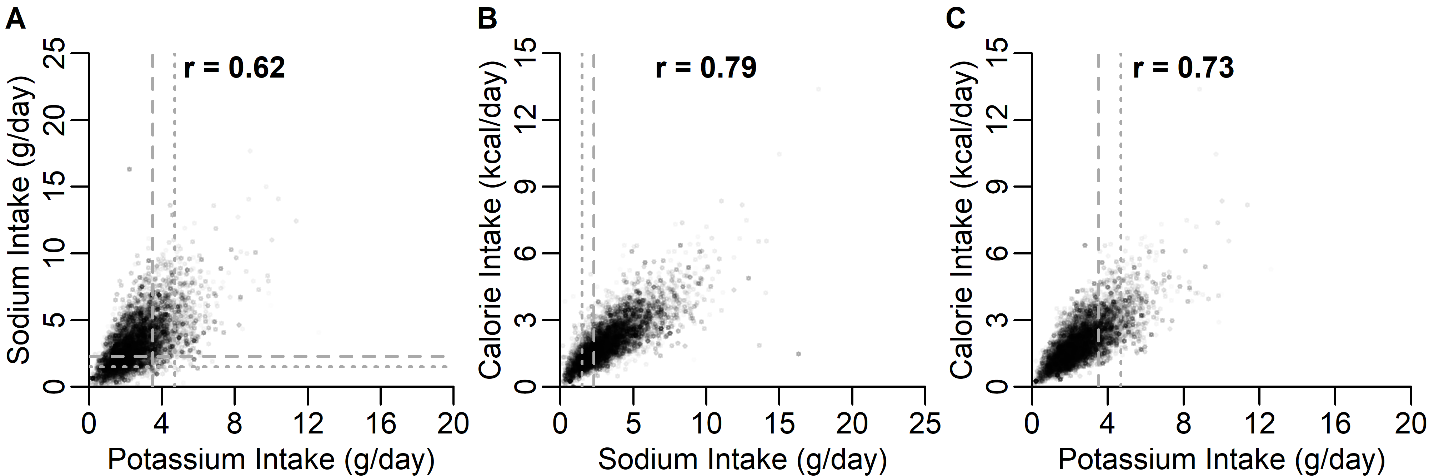


Figure S3: Dietary patterns for black adults with hypertension in the United States showing the relationship between daily (A) potassium and sodium intakes, (B) sodium and calorie intakes, and (C) potassium and calorie intakes. Dashed and dotted lines represent recommended and ideal intakes, respectively. Pearson correlation coefficients are provided.


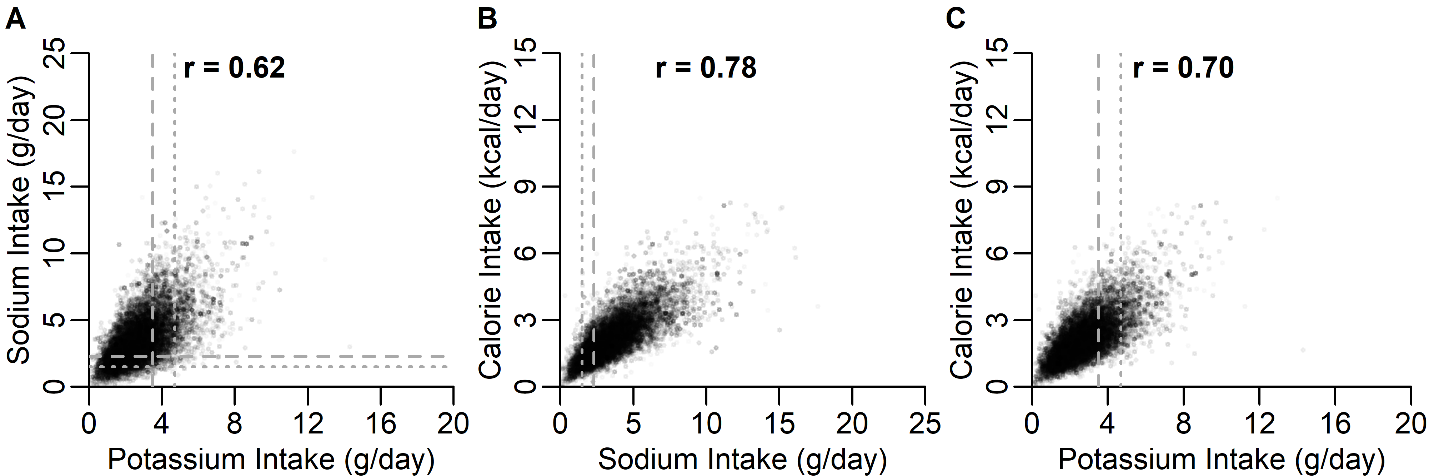


Figure S4: Dietary patterns for white adults with hypertension in the United States showing the relationship between daily (A) potassium and sodium intakes, (B) sodium and calorie intakes, and (C) potassium and calorie intakes. Dashed and dotted lines represent recommended and ideal intakes, respectively. Pearson correlation coefficients are provided.


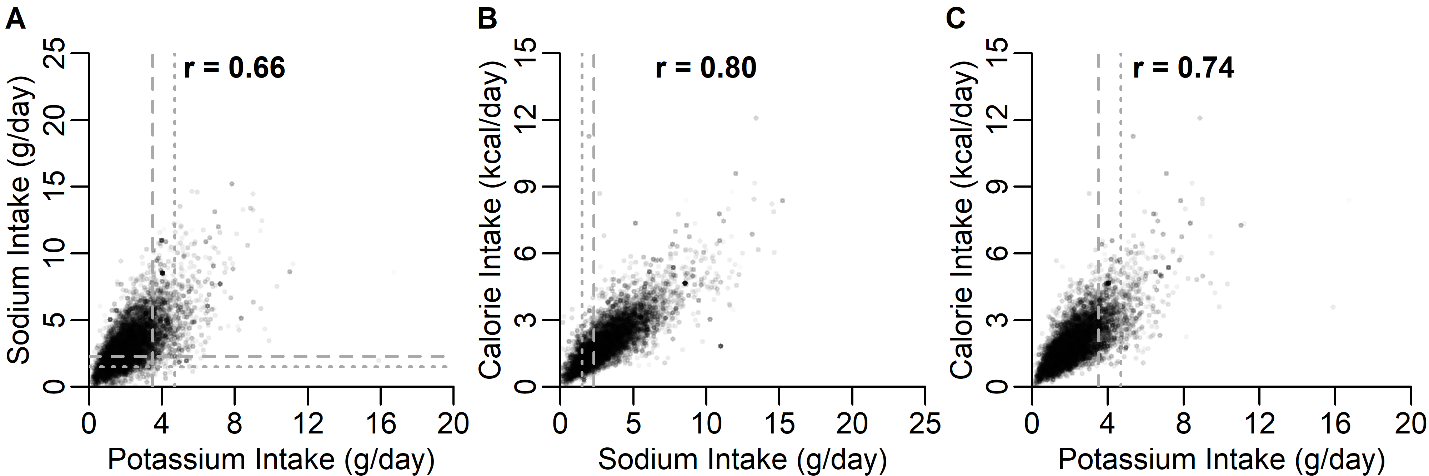


Figure S5: Dietary patterns for Hispanic adults with hypertension in the United States showing the relationship between daily (A) potassium and sodium intakes, (B) sodium and calorie intakes, and (C) potassium and calorie intakes. Dashed and dotted lines represent recommended and ideal intakes, respectively. Pearson correlation coefficients are provided.


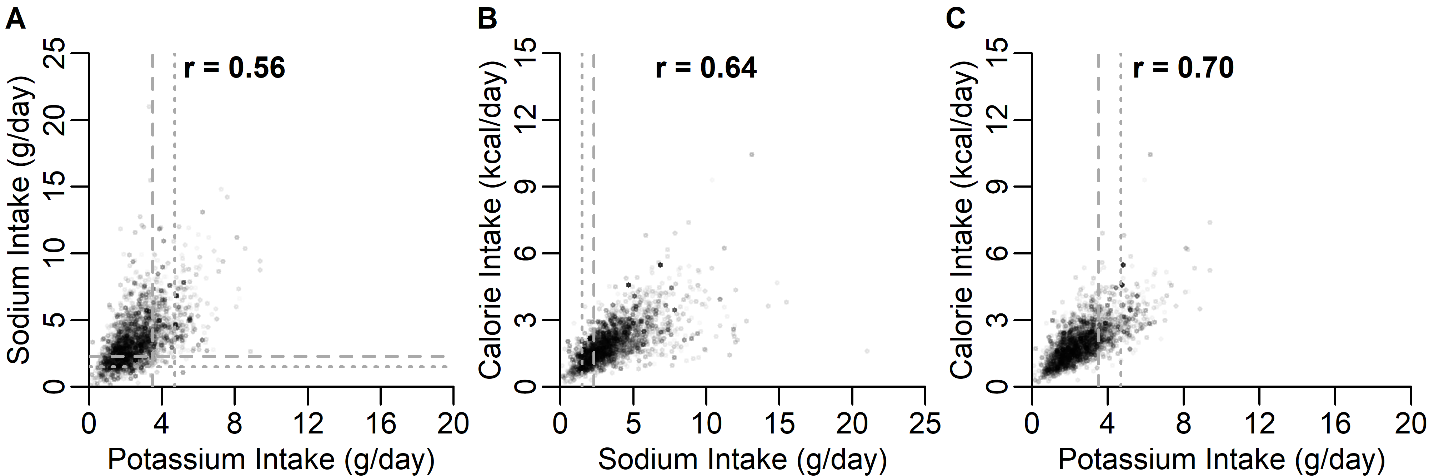


Figure S6: Dietary patterns for adults of other races/ethnicities with hypertension in the United States showing the relationship between daily (A) potassium and sodium intakes, (B) sodium and calorie intakes, and (C) potassium and calorie intakes. Dashed and dotted lines represent recommended and ideal intakes, respectively. Pearson correlation coefficients are provided.
